# What do caregivers of Veterans with dementia feel unprepared for: Results from the multisite HERO CARE caregiver survey

**DOI:** 10.1002/alz.095466

**Published:** 2025-01-09

**Authors:** Roshni Singh, Marianne E Desir, Richard A Munoz, Saanvi Lamba, Diana I Ruiz, Pranjal Tyagi, Sandra Garcia‐Davis, Stuti Dang

**Affiliations:** ^1^ University of Miami Miller School of Medicine, Miami, FL USA; ^2^ Miami Veterans Affairs Healthcare System, Miami, FL USA; ^3^ Geriatric Research, Education and Clinical Center, Miami, FL USA; ^4^ South Florida VA Foundation for Research and Education, Miami, FL USA; ^5^ Flinthill school, Oakton, VA USA; ^6^ University of Miami, Miami, FL USA

## Abstract

**Background:**

Veterans with dementia often experience a wide range of multidomain needs, many of which are met by informal caregivers. Yet, caregivers are often not adequately prepared to address the needs of the dementia patient. This study aimed to identify caregiving responsibilities for which informal caregivers feel insufficiently prepared.

**Method:**

We used round one data from the HERO CARE Survey, a prospective multisite survey administered to assess unmet needs of Veterans and caregivers, with oversampling of Veterans with a higher predicted 2‐year long‐term institutional care risk. For the current study we focused on the responses of caregivers of Veterans with dementia to the following survey questions: “Out of all the tasks that you help the Veteran with, how prepared do you feel to do these tasks?” and “Is there anything specific you would like to be better prepared for?” Content analysis was completed using the responses to the second of these questions, which was open‐ended.

**Result:**

The survey responses of 256 caregivers were eligible for the current study. The included caregivers were 71.3 (SD: 10.9) years old on average, 89.1% female, 68% spousal caregivers, 73.0% non‐Hispanic White, 86.3% primary caregivers, and 55.5% had provided care for over 5 years. Of the caregiving tasks they had to help the Veteran with, 2% felt unprepared, 55.9% somewhat prepared, and 42.2% very prepared to do the tasks. Caregivers’ most reported concerns related to their caregiving skills included addressing difficult behaviors (15.2%) and providing assistance with ADLs (14.5%); related to increasing ADL and IADL needs ‐ ADLs (18.4%) and IADLs (5.9%); related to information/knowledge included dementia stages and what to expect (10.9%), managing finances/legal (9.0%), and resources and care options for long‐term care and placement (5.5%).

**Conclusion:**

Many caregivers of Veterans with dementia feel less than fully prepared for their caregiving responsibilities. They may benefit from increased training and support related to addressing difficult behaviors, ADL/IADL assistance, knowing what to expect as dementia progresses, managing financial and legal concerns in the setting of dementia, and understanding options related to long‐term care and placement.

